# Professional football training and recovery: A longitudinal study on the effects of weekly conditioning session and workload variables

**DOI:** 10.1371/journal.pone.0310036

**Published:** 2024-09-10

**Authors:** Davide Curzi, Stefano Amatori, Fioretta Silvestri, Lorenzo Marcelli, Matteo Campanella, Fabrizio Perroni, Maria Chiara Gallotta, Alessandra Favoriti, Carlo Baldari, Laura Guidetti

**Affiliations:** 1 Department of Humanities, Movement and Education Sciences, University "Niccolò Cusano", Rome, Italy; 2 Department of Biomolecular Sciences, University of Urbino Carlo Bo, Urbino, Italy; 3 Department of Theoretical and Applied Sciences, eCampus University, Novedrate, Italy; 4 Department of Physiology and Pharmacology “Vittorio Erspamer”, Sapienza University of Rome, Rome, Italy; 5 Ternana Football Club, Terni, Italy; Portugal Football School, Portuguese Football Federation, PORTUGAL

## Abstract

The main purpose of this longitudinal study was to investigate football players’ recovery status, through hormonal response, in relation to accumulated workload at two comparable time points of the first (T1) and second half (T2) of the competitive season. Moreover, this study investigated athletes’ hormonal response to a typical weekly conditioning session (5 days before match: MD-5), at T1 and T2, to detect changes in players’ recovery capability over time. Salivary cortisol (sC) and testosterone (sT) of 24 professional players (27.8 ± 4.1 years of age) were collected before, after, and 24 hours following MD-5 in two comparable microcycles of T1 and T2. GPS training data (total and high-intensity distance) of the 7 and 28 days before sampling were used to obtain athletes’ acute and chronic workloads. Results showed a pre-training significant decrease of sT and an increase of sC (p<0.05) in T2, compared to T1. Moreover, athletes showed high sC and low sT levels before, after and 24 hours following MD-5 in T2. Workload analysis revealed significant correlations of chronic load with sC (r = 0.45, p = 0.056) and T/C ratio (r = -0.59; p = 0.007). These results suggested that, in professional football, chronic workload has a greater impact on players’ recovery time than acute workload over the sport season. Moreover, athletes’ hormonal response to the weekly conditioning session at T2 revealed an altered anabolic/catabolic balance, highlighting the key role of continuous internal and external workload monitoring during the season.

## Introduction

The congested match schedule and the short recovery periods represent a great challenge for professional football athletes, often leading to stressful conditions [1, 2]. In fact, the physical and technical demands at the elite level have changed in the last years, and players need to be prepared to sustain training and matches involving a high metabolic and neuromuscular cost [3]. Longitudinal studies on professional football players revealed that high-intensity distance covered and sprint actions performed significantly increased during matches [4–6].

A full physiological recovery from a match would take at least three days since muscle function impairment and injury-related markers are still evident 72 hours after matches [7]. However, due to the tight match schedule, athletes may get into training while still recovering/regenerating [7]. In the long-term scenario, accumulated workload without optimal recovery could also result in a higher perception of muscle soreness, fatigue [8], stress [9] and impaired performance [10], affecting athletes’ mental and physical health and increasing injury risk [11]. For the abovementioned reasons, athlete recovery monitoring has become essential to have reliable information on players’ health status and readiness for competition.

In order to monitor athletes’ external training loads, GPS data of acute (7 days) and chronic (28 days) workloads could be used to analyse specific periods of training within the season. Acute workload could greatly affect athletes’ performance since it is chronologically close to the testing or match day, while chronic workload could give important information on athletes’ exposure to fatigue and stress in a specific seasonal range. Moreover, the ratio between them (A:C) could be useful to manage the progression in training load and has been used in several studies [12–14].

The internal workload is instead associated with the individual physiological response to training [15] and could be also measured by the rate of perceived exertion (RPE) [16] and endocrine marker variations [17]. Among hormones, cortisol and testosterone play a major role in immune and metabolic responses to stressful conditions and are commonly used as biomarkers of exercise-induced effects [18–20]. The cortisol level tends to increase after high-intensity exercise bouts [21], and it seems to impair performance throughout post-match recovery due to enhanced catabolic processes [22]. Conversely, testosterone is thought to counterbalance cortisol actions and is frequently shown to decrease or remain unchanged after matches [22]. Moreover, the testosterone to cortisol ratio (T/C) is commonly used to evaluate organism response to acute and chronic physical stressors [17, 23]. The T/C ratio is considered an index of anabolic/catabolic balance and seems to reflect the recovery status after training or competition [18]. A decrease in T/C ratio could be associated with fatigue states and its monitoring could be useful to prevent athletes from overtraining. These hormonal changes during the sports season are still discussed in literature, since their fluctuations in response to training load are not clear. In fact, some studies described different trends for cortisol and testosterone during both pre-season [24, 25] and competitive phases [26, 27].

Therefore, cortisol and testosterone levels in football players during a competitive season need to be further investigated to gain a better understanding of athletes’ response to acute and chronic workload and their readiness for competition.

With the aim to compare athletes’ physiological response to the workload accumulated at two comparable time points of the season, the first aim of this study was to investigate the hormonal profile of athletes in the middle of the first and second half of the football championship in relation to the training load variables. Furthermore, to study the impact of the same weekly microcycle on the recovery status of athletes in the first and second half of the season, the second aim of this study was to analyse the hormonal changes of athletes following the weekly conditioning session (first training session of the week with the highest training load volume) of two comparable microcycles. To our knowledge, this is the first work which analyses the relationship between workload variables and players’ recovery status, across the hormonal response, at the specific time points previously described. The results of this work, in addition to expanding current knowledge on player recovery in professional football, could have a practical value for microcycles’ design in order to optimize athletes’ performance and reduce injury risk.

## Materials and methods

### Participants

Twenty-four professional football players (mean age 27.8 ± 4.1 years; body mass index 23.0 ± 1.3 kg/m^2^), starters and no-starters, were included in this study. All players belonged to the roster of the Ternana Football Club, competing in the BKT championship, the second-highest division of the Italian football league system. To be eligible, players were required to be healthy and without any physical or psychological difficulties that could affect the study (during the whole sport season the athletes were monitored weekly by the club’s medical staff). The following exclusion criteria were applied: (a) missing more than 10% of training sessions during the sport season; (b) taking any medical therapy, nutritional supplements or drugs that could compromise data collection.

After having informed athletes about the project with the aid of a written document, the informed consents were collected prior to the beginning of the study. Ethical approval was granted by the Institutional Review Board of the University Niccolò Cusano, study protocol number MO3/22 in accordance with the 1964 Helsinki Declaration and its later amendments or comparable ethical standards.

### Study design

The study was conducted during the first 8 months of the BKT championship 2022/2023. The twenty teams’ tournament was composed of thirty-eight matches (nineteen for each half of the season). Athletes were assessed at two comparable time points: 11 weeks after the beginning of the first half of the season (T1) and 11 weeks after the beginning of the second half of the season (T2). In T1 and T2 weekly microcycles the athletes played against the same opposing team. The latter showed a high percentage of the same athletes on the field between the two matches, as well as a comparable playing style. Furthermore, the two weekly plans of the analysed team were comparable to each other (Table 1).

**Table 1:** The weekly training plan for T1 and T2 microcycles.

Weekly Plan	Morning sessions	Afternoon sessions
MD-6	Day Off	
MD-5	Off	Conditioning Session
MD-4	Off	Soccer Training
MD-3	Soccer Training	Off
MD-2	Soccer Training	Off
MD-1	Light Soccer Training	Off

Match day

Both testing days corresponded to the “-5 training session” (5 days before the match day: MD-5), which is the conditioning session of the weekly microcycle of periodization. In both halves of the season, the MD-5 began at 3:30 pm and consisted of different phases: a general warm-up (≈15/20 min), interval training exercises (≈ 16/18 min), technical-tactical training (≈ 45 min), running progressions (≈ 20 min, carried out in the first half only) and a final match (≈ 30 min in the first half, ≈ 15 min in the second half).

**Fig 1:**
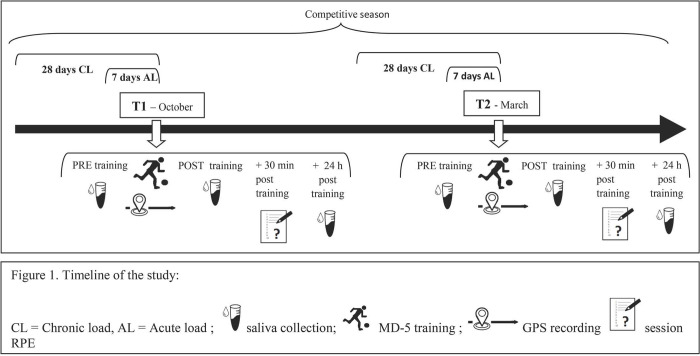
Timeline of the study.

Salivary samples, GPS data and RPE scores were collected in order to obtain athletes’ hormonal concentrations, workload data, and perceived effort scores, respectively. The timeline of the study design is described in Fig 1.

### Saliva collection

To collect saliva samples, athletes were asked to avoid food, sports drinks and food supplements for at least 2 hours before the beginning of the MD-5 training session. In addition, they were asked to rinse their mouth with water before withdrawing. Saliva samples were collected before, after (within 10 minutes after the end of the training session) and 24 hours after the end of MD-5. The training session of the following day was scheduled in the afternoon in order to obtain hormonal data on effective 24 hours recovery. To minimize circadian rhythm effects, all samplings were carried out in the same daily time slot. Salivary samples were collected with a cotton swab and saliva collecting tube (Salivette Sarstedt ^®^), centrifuged at 3000 rpm for 10 min with a temperature of RT°C and stored at -20°C. Using a Testosterone and Cortisol Saliva ELISA kit (Tecan), concentrations of sT and sC were obtained according to their production protocols.

The testosterone limit of quantitation (LoQ) corresponded to 2.1 pg/ml, while Cortisol LoQ was 0.003 μg/dl. The intra-assay coefficient of variation for testosterone and cortisol was 5.6% and 4.3% respectively, while the inter-assay coefficient of variation was 8.7% for testosterone and 13.2% for cortisol.

### Internal and external training load

To measure internal training load, 30 minutes after the end of the training sessions, athletes were asked to rate their RPE by means of Borg’s CR-10 rating scale [28].

In order to assess external training load, during the whole season, players were monitored by means of K-shirt, an integrated detection system, characterized by GPS K-50 (recording frequency 50 Hz, K-Sport, Montellabate, Italy), able to track heart rate second by second. Every training and match workload data has been recorded. During match days, compensatory works of non-starter players have been included in data collection. Total distance (TD) and high-intensity distance (≥ 16 km/h; HID) were considered as indices of training load volume and intensity, respectively. The total load (TL) was calculated for each player as the sum of each training session or match completed over two-time windows: 7 days, representing an ‘acute’ load, and 28 days, representing a ‘chronic’ load. Moreover, in order to consider variations in the way the loads were accumulated, an exponentially weighted moving average (EWMA), which assigns a decreasing weight for each older training load value, was calculated as previously described [29]. The ratio between the acute (7d) and the chronic (28d) EWMA_TD_ and EWMA_HID_ were calculated in order to describe variations in the training loads (A:C ratio).

### Statistical analysis

Data were reported as mean ± standard deviation or median [first and third quartile] (if not normally distributed). The normality distribution was checked for each dependent variable by exploring skewness and kurtosis values. Non-normally distributed variables (sC and T/C ratio) were treated by applying a log transformation, as suggested by Miller & Plessow [30]. Unpaired Student’s t-tests were used to verify the difference in training loads and salivary measures between the two halves of the season. Pearson’s correlations were computed for each half in order to verify if training load measures would associate with salivary hormone values in the pre-training measurements. Coefficients of correlations were interpreted as follows: *r* ≤ 0.20: very weak, 0.20–0.39: weak, 0.40–0.59: moderate, 0.60–0.79: strong, ≥ 0.80: very strong. In order to test the effect of the training session and the period of the season on the dependent variables (sT, sC and T/C ratio), a linear mixed-effects model was computed with the *lmer* function, using time (pre, post and 24h post-training), half (first or second) and their interaction (time×half) as fixed effects, while participants’ ID was included in the model as a random factor, in order to account for the repeated measure nature of the data. Post-hoc tests were also computed to test the time effect on the two halves of the season separately. Analyses were performed using RStudio (v. 2023.06.0+421, Posit), at a standard significance level of 0.05.

## Results

### Effects of accumulated workloads on players’ recovery status

**Fig 2:**
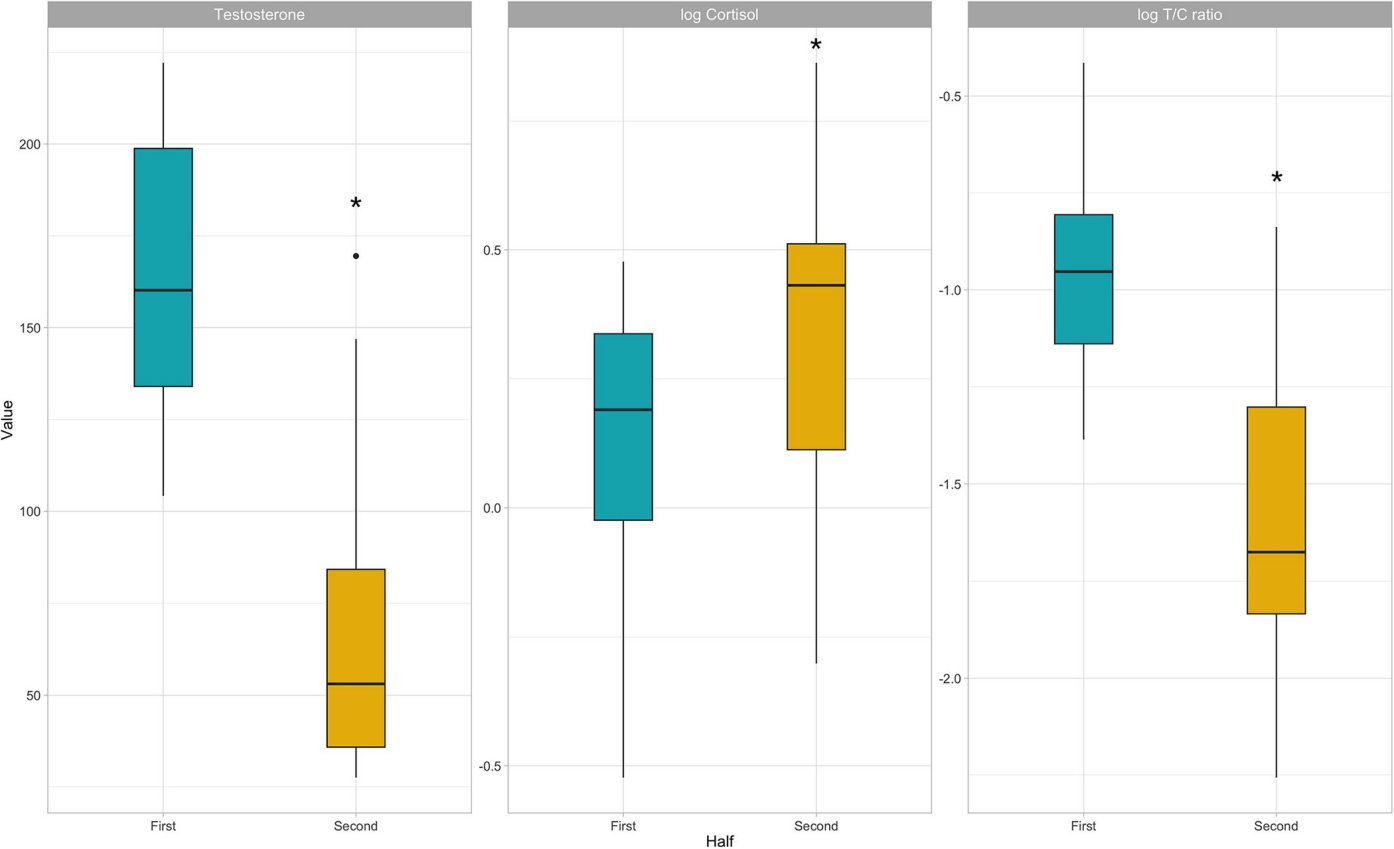
Box plots represent salivary measures in the first and second half of the season.

All pre-training session salivary measures in the second half of the season showed significant differences compared with the first half of the season (p<0.05), with reduced sT and T/C, and increased sC levels (Fig 2).

Among the training load variables, no differences were reported for the total load over 7d and 28d, while significant differences between the two halves of the season were found for the EWMA and A:C values, except for the EWMA of the HID (Table 2).

**Table 2:** Descriptive statistics (mean ± standard deviation) of training characteristics before T1 and T2, respectively.

	T1—First half	T2—Second half	*p*(t)
**TD 7d [m]**	23667 ± 4240	23992 ± 7126	0.855
**HID 7d [m]**	3548 ± 960	3111 ± 1102	0.173
**TD 28d [m]**	105961 ± 24199	113344 ± 17604	0.262
**HID 28d [m]**	17671 ± 5675	15883 ± 3266	0.220
**EWMA TD 7d [m]**	2364 ± 798	3728 ± 965	**< 0.001**
**EWMA HID 7d [m]**	384 ± 131	504 ± 157	**0.009**
**EWMA TD 28d [m]**	2812 ± 721	3416 ± 619	**0.005**
**EWMA HID 28d [m]**	451 ± 137	461 ± 115	0.805
**A:C TD**	0.81 ± 0.23	1.07 ± 0.19	**< 0.001**
**A:C HID**	0.84 ± 0.30	1.08 ± 0.21	**0.005**

TD, total distance; HID, high-intensity distance; EWMA, exponentially weighted moving average; A:C, acute workload/chronic workload ratio.

Correlation analysis was performed separately for the first and second half data; full correlations matrix is reported as Supplementary Material (S1 Table in S1 File). Results showed no associations between acute training loads (TD_7d_ and HID_7d_), neither for sT or sC values (*r* ranging from -0.22 to 0.23, *p*>0.05), in none of the two halves of the season. Conversely, moderate associations were found between chronic training loads and sC in the second half of the season (TD_28d_: *r* = 0.45, *p* = 0.056). Coherently, moderate negative correlations were also reported between chronic training loads and T/C ratio in the second half of the season (TD_28d_: *r* = -0.59, *p* = 0.007; HID_28d_: *r* = -0.42, *p* = 0.077; EWMA TD_28d_: *r* = -0.52, *p* = 0.022). In addition, the A:C ratio of HID seemed to be associated with sT levels in the first half (A:C_HID_: *r* = 0.58, *p* = 0.012) and with the T/C ratio in the second half of the season (A:C_HID_: *r* = 0.54, *p* = 0.016).

### Effects of MD-5 on salivary hormone kinetics

In both halves of the season, players performed a comparable microcycle with a conditioning MD-5 training session (see Methods for a detailed description of the session). The total distance completed in the training session was 11020 ± 3022 m for the first half, and 8294 ± 1193 m in the second half of the season, with 2314 ± 887 m and 1200 ± 222 m performed at high intensity, respectively. The team average RPE scores following the MD-5 in the first and second halves of the season were 7.4 and 7.0, respectively.

**Fig 3:**
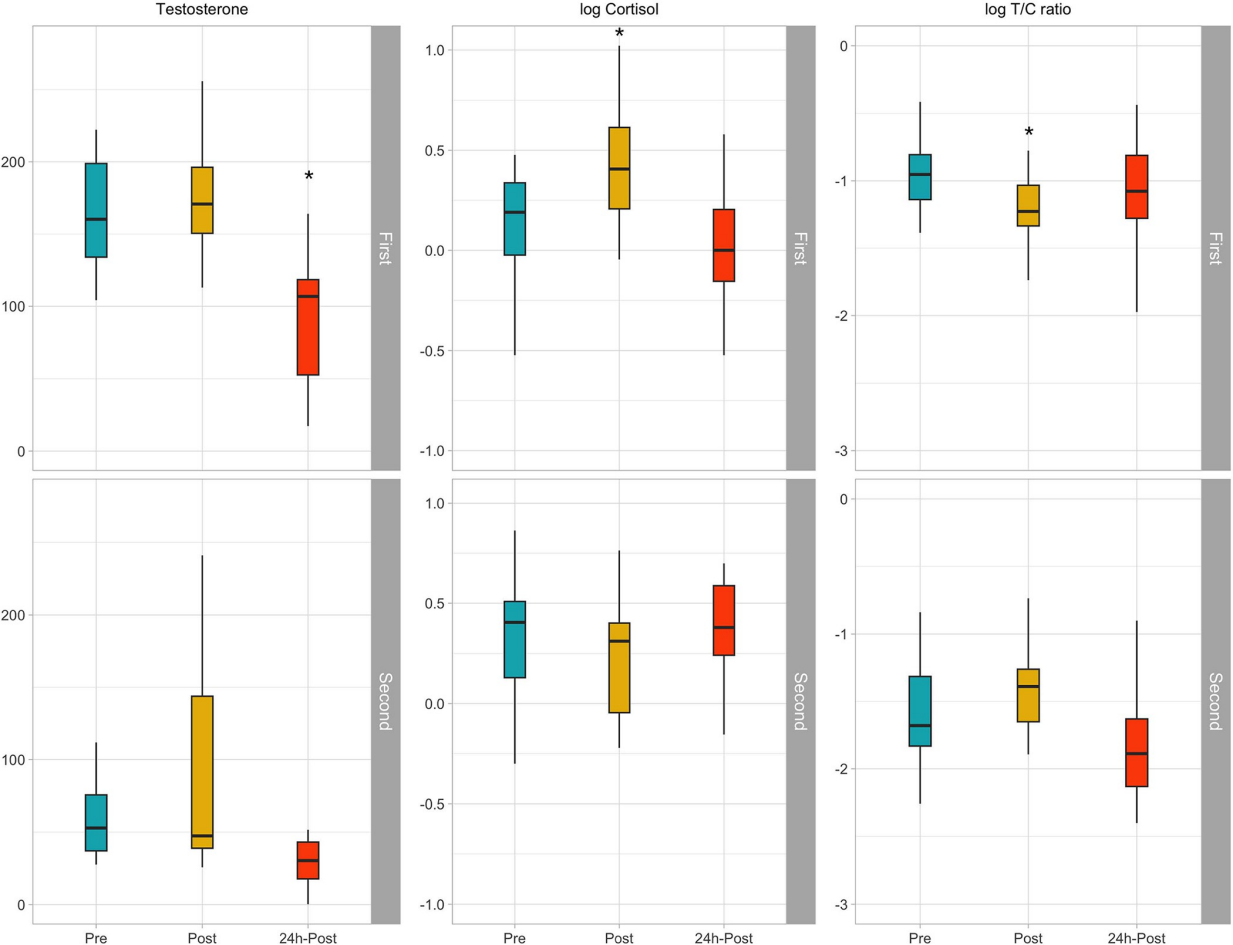
Boxplots representing variations between pre-, post- and 24h-post training sessions, for the salivary measures, in the first (above) and second (below) half.

Results showed a significant effect of the half on all the dependent variables, except sC, with higher sT and T/C ratio, and lower sC values in the first half of the season, at all time points. Regarding sT, a significant effect of time was reported (*F* = 16.3, *p*<0.001), showing a significant reduction at 24-h post-training in the first (*p*<0.001), but not in the second half of the season (*p* = 0.074). Interestingly, sC showed a different trend in the two halves of the season (time×half interaction: *F* = 7.97, *p* = <0.001): while in the first half, it significantly increased in the post-training compared to the basal value (*p* = 0.006), in the second half of the season sC showed notably high levels already in the pre-training, and then slightly decreased in the post-training, although not significantly (*p* = 0.112). In both halves of the season, data were not different from pre-training levels at 24h. As expected, the T/C ratio also showed a significant time (*F =* 5.27, *p =* 0.006) and time×half effect (*F* = 6.13, *p* = 0.003), with the T/C ratio in the first half which significantly reduced in the post-training (*p* = 0.034), while no significant changes were reported in the second half of the season. All the data are reported as median [first and third quartile] in Table 3 and graphically in Fig 3.

**Table 3:** Descriptive statistics (median [first-third quartile]) grouped by Half (first vs second half) and Time (pre-, post- and 24h post-training).

	First half			Second half					
	Pre-training	Post-training	Post-24h	Pre-training	Post-training	Post-24h	Time	Half	Time × Half
**Testosterone** **[**pg/ml]	160.20 [134.00–198.80]	170.75 [150.50–196.18]	106.80 *** [52.70–118.40]	52.82 [37.00–75.48]	47.33 [38.80–143.78]	30.27 [17.60–43.06]	**< 0.001**	**< 0.001**	0.271
**Cortisol [**ng/ml]	1.55 [0.95–2.18]	2.55 ** [1.63–4.13]	1.00 [0.70–1.60]	2.55 [1.35–3.23]	2.05 [0.90–2.53]	2.40 [1.75–3.90]	0.486	**0.042**	**< 0.001**
**T/C ratio**	0.11 [0.07–0.16]	0.06 * [0.05–0.09]	0.08 [0.05–0.15]	0.02 [0.01–0.05]	0.04 [0.02–0.06]	0.01 [0.01–0.02]	**0.006**	**< 0.001**	**0.003**

Note: * significant difference from pre-training of the same half (reference category)

*: p<0.05

**: p<0.01

***: p<0.001.

## Discussion

### Players’ physiological status at the middle of the first and the second half of the competitive season

In the second half of the season, players showed a significantly decreased level of sT and T/C ratio, and a significantly increased level of sC compared to the first half of the season. In agreement with our results, analysing 30 professional football players during a competitive season, Handziski and colleagues [26] found an increase in cortisol levels and a decrease in testosterone values in the blood at the end of the season if compared to the levels at the beginning and mid-season. Moreover, at the end of the season, the authors found a decrease of more than 30% in the T/C ratio with respect to the beginning values, suggesting a possible hormonal dysfunction [26]. However, the increased catabolic and the decreased anabolic trend during elite football season is still discussed in the literature. In fact, a significant decrease in testosterone levels [31, 32], as well as a significant increase [33] or no variations [34] over a competitive season, have been previously described. The testosterone increase during the season was explained by the authors themselves [33] with low starting testosterone levels, reflecting a pre-season catabolic state. Changes in cortisol concentration during the football season are also discussed in the literature. Some authors found that cortisol levels significantly increased in the mid-season period compared to pre-season values [31, 34]. On the contrary, no significant cortisol changes have been found by Saidi et al. [32] and Andrzejewski et al. [35] after 3 and 6 months of football training, respectively. However, in both studies, the first evaluation was carried out in the middle [35] and in the last [32] part of the sports season, when athletes might have already reached a high value of stress. Regarding T/C ratio response, Saidi and colleagues [32] studied blood cortisol and testosterone variations during 12 weeks of a competitive season in elite football athletes finding a significant decrease in T/C ratio with the increase in match frequency. In agreement with our results, these findings suggest a progressive phase of anabolic impairment and catabolic predominance in relation to the accumulated workload. In disagreement with our data, Michailidis and Yiannis [31] did not detect any T/C ratio modulation in football elite players during sport season. In fact, although the authors found a significant decrease in testosterone levels, confirming our results, they also detected decreased cortisol levels during the season.

In summary, even if several studies agreed with us in describing a catabolic environment in response to football training or matches, conflicting results appeared regarding the single variations of cortisol and testosterone over a competitive season. Anyway, the analysis of these hormones and their ratio is crucial to investigate player recovery, since if this anabolic-catabolic trend is confirmed, the football players are likely to experience a reduction in performance during the season [33].

### Correlation between training load variables and players’ recovery status

Both acute and chronic load measures suggested similar workload values in the two analysed periods (Table 2), according to existing literature [36]. Even if athletes were exposed to a comparable amount of training, our findings pointed out how indicators of stress (higher sC) and catabolism mechanisms (lower T/C ratio) appeared in the second part of the season. In agreement with our results, some authors found that athletes could experience increased levels of fatigue and stress at the end of the season despite having trained with similar [37] or lower workloads [12] compared to the early season. Although we found no differences in total acute and chronic workloads, our analysis revealed that EWMA and the A:C ratio in the second half of the season were significantly higher than in the first half. These results suggest a different load distribution in the second part of the season, with minimal changes in total acute and chronic load. A high A:C ratio, calculated with EWMA was also significantly associated with an increased injury risk [13, 38]. In our study, the injury rate during the season was not measured, but the decrease in T/C ratio in the second half of the season reflects a situation of increased stress, which could be also related to a higher injury risk. In the second half of the season, moderate negative correlations between chronic training loads and T/C ratio were found, and coherently, at this time point cortisol levels showed a moderate positive correlation with chronic loads, although not significant (*p* = 0.056). These results suggested that athletes’ endocrine response is more affected by chronic than acute workload. These results partially confirmed those obtained by Rowell and colleagues [39], who concluded that athletes’ hormonal response to training appears to be impacted by both very short (3-day) and relatively long (28-day) preceding workloads.

In agreement with our findings, Silva et al. [40] revealed that match exposure was positively correlated with cortisol levels and negatively correlated with T/C ratio in the mid-season. On the contrary, other authors found that testosterone and cortisol were not correlated with accumulated workload in adolescent football players [41]. However, the hormonal response can be also influenced by physiological fluctuations during adolescence [42].

Further results of the present study revealed how the A:C ratio of HID (distance covered ≥ 16 km/h) was positively correlated with testosterone values in the first half, and with the T/C ratio in the second half of the season. These findings suggested an elevated testosterone concentration in response to acute high-intensity workload, in agreement with previous studies [43]. Concerning cortisol response to high-intensity workload, in disagreement with previous research [44, 45], the results of this study did not show a significant association between HID and sC levels.

### Analysis of two comparable microcycles of the season: players’ recovery status after MD-5 conditioning session

Although the load volume and intensity of MD-5 in the second half of the season were lower than those in the first half, the sC level 24 hours after training decreased in the first half of the season but not in the second, suggesting a longer recovery time in the second period if compared to the first one. Furthermore, pre-training sC levels in the second half of the season appeared so high that they were similar to post-training sC levels in the first half. These results seemed to suggest a chronic catabolic state of the players in the second half of the season.

Concerning testosterone response to training sessions, while sT levels significantly decreased at 24 h post-training in the first half of the season, in the second half, results did not show significant differences compared to pre-training values. Moreover, in the second half of the season, the sT levels were found to be low at pre-, post- and 24 hours after training session. These findings, according to the sC response, confirmed the prevalence of the catabolic state on the anabolic one in the second half of the season. As a consequence of the hormonal variations previously described, the T/C ratio decreased post-training in the first, but not in the second half of the season, when the ratio was already low at pre-training measurement.

Rowel and colleagues [46] found an increase in both cortisol and testosterone levels 30 minutes after the match during the pre-season period. On the contrary, Peñailillo and colleagues [47] observed a decrease in testosterone levels and T/C ratio after a friendly international match, according to our first half results. In studies carried out during the second half of a football season, some authors observed a significant increase in sC levels 30 minutes [45] and 24 h [48] after a competitive match.

Handziski et al. [26] analysed 30 football athletes before and after the pre-season period, as well as at the end of the competition phase, without finding significant differences in the acute hormonal response to a training session. Unfortunately, the authors used a maximal test as a training stimulus, which presents workloads incomparable to those of our proposal. However, in agreement with our results, at the end of the season, an increased cortisol level was found even before the maximal test, describing a chronic catabolic state of the athletes at the end of the competitive phase.

In summary, while the increase in sC levels and the decrease in sT levels after exercise in the first half of the season are in agreement with those described in the literature, the findings of minimal or no hormonal changes in the second half of the season may reflect a state of chronic fatigue. For this condition, the athletes may have been characterized by sT levels so low to not allow further decreases, and by sC values so high not to allow further increases. In fact, behavioural science theories report that in one-time acute stress condition, the increase in cortisol levels reflects an adaptive physiological response, while in chronic stress situations, there is a decrease in cortisol response [49].

### Limitations of the study

A regular season of elite soccer is characterized by the turnover of athletes (injuries, transfer market,…), therefore, in the two analysed time points of the sports season, a limited number of players from the full roster was evaluated.

A possible limitation that should be acknowledged is the use of the A:C ratio as a descriptive measure of training load, given the various criticisms associated with its use. Potential methodological issues include the mathematical nature of the ratio itself, the coupling of acute and chronic timeframes, and the variability introduced by different training load variables and time windows. Additionally, most critiques focus on its application as a prognostic tool for injury prediction, whereas in our study, it was employed for descriptive purposes alongside other absolute training load measures. Nevertheless, while these limitations should be carefully considered when interpreting the A:C ratio data, its use offers an interesting perspective on training load distribution over time.

In this study, specific tests on players’ psychological status were not carried out, however, the psychological environment during the two included time points seemed comparable. In fact, analysing external factors which could have influenced psychological status, such as team ranking and the score of the last matches before testing, a positive or negative trend is not revealed. Future research should introduce psychological measurements to better investigate hormonal variation during the season.

## Conclusion

In conclusion, this research shows the significant impact of chronic workloads on the hormonal response of professional football players during the sport season, suggesting that chronic workload assessment could help in players’ recovery management more than acute workload evaluation. In addition, hormonal responses after the weekly conditioning session suggested that in the first half of the season, when athletes exhibit a good hormonal balance, they showed an acute physiological response to training, with changes in sT, sC levels and their ratio. On the contrary, at the end of the season, when athletes showed an altered anabolic/catabolic ratio, their capability to respond to training stimuli was impaired. While on the one hand, the catabolic state found in the second part of the sports season does not necessarily characterize all professional football teams, on the other, the literature demonstrates that it is not a one-time event. Suggesting a lack of full recovery, an altered anabolism/catabolism balance could hurt maintaining performance and it could increase the risk of injury. The findings of this manuscript suggest the need for a frequent assessment of chronic load and periodic monitoring of athletes’ hormonal responses for coaching and medical staff, respectively. Moreover, these results could represent a starting point for investigating the effects of different management of microcycle building [50] on football players’ recovery status.

## Supporting information

S1 File Correlation matrix between training load and salivary measures. Pearson’s correlation (r) and p-values (two-tailed) are reported; values highlighted in bold are those statistically significant (p<0.05).
(DOCX)

S2 File Training and salivary data set. (XLSX)
