# Supplementary material for: Professional football training and recovery: A longitudinal study on the effects of weekly conditioning session and workload variables
Source: PLoS One. 2024 Sep 10;19(9):e0310036. doi: 10.1371/journal.pone.0310036 (PMC11386442; doi:10.1371/journal.pone.0310036)
Supplement: S1 File — Pearson’s correlation (r) and p-values (two-tailed) are reported; values highlighted in bold are those statistically significant (p<0.05). (DOCX) [file pone.0310036.s001.docx]

**Supplementary Material 1.** Correlation matrix between training load and salivary measures. Pearson’s correlation (r) and p-values (two-tailed) are reported; values highlighted in bold are those statistically significant (p<0.05).

|  |  | **Testosterone** | | **Log 10 Cortisol** | | **Log 10 T/C ratio** | |
| --- | --- | --- | --- | --- | --- | --- | --- |
|  |  | *First half* | *Second half* | *First half* | *Second half* | *First half* | *Second half* |
| **TD 7d** | *Pearson’s r* | 0.014 | -0.219 | 0.071 | 0.23 | -0.079 | -0.351 |
|  | *p-value* | 0.955 | 0.368 | 0.780 | 0.344 | 0.755 | 0.140 |
| **HID 7d** | *Pearson’s r* | 0.216 | 0.048 | 0.103 | 0.180 | -0.023 | -0.148 |
|  | *p-value* | 0.390 | 0.844 | 0.683 | 0.462 | 0.927 | 0.545 |
| **TD 28d** | *Pearson’s r* | -0.357 | -0.212 | -0.359 | 0.445 | 0.238 | **-0.593** |
|  | *p-value* | 0.146 | 0.384 | 0.143 | 0.056 | 0.341 | **0.007** |
| **HID 28d** | *Pearson’s r* | -0.330 | -0.059 | -0.109 | 0.35 | -0.004 | -0.415 |
|  | *p-value* | 0.182 | 0.810 | 0.666 | 0.142 | 0.987 | 0.077 |
| **EWMA TD 7d** | *Pearson’s r* | 0.098 | -0.203 | 0.010 | 0.312 | 0.020 | -0.442 |
|  | *p-value* | 0.700 | 0.404 | 0.969 | 0.193 | 0.939 | 0.058 |
| **EWMA TD 28d** | *Pearson’s r* | -0.221 | -0.211 | -0.213 | 0.379 | 0.131 | **-0.523** |
|  | *p-value* | 0.379 | 0.386 | 0.396 | 0.110 | 0.605 | **0.022** |
| **EWMA HID 7d** | *Pearson’s r* | 0.246 | 0.073 | 0.243 | 0.180 | -0.156 | -0.161 |
|  | *p-value* | 0.325 | 0.768 | 0.332 | 0.460 | 0.536 | 0.510 |
| **EWMA HID 28d** | *Pearson’s r* | -0.221 | -0.031 | 0.038 | 0.311 | -0.117 | -0.357 |
|  | *p-value* | 0.378 | 0.899 | 0.882 | 0.195 | 0.645 | 0.133 |
| **A:C ratio TD** | *Pearson’s r* | 0.301 | -0.124 | 0.227 | 0.124 | -0.125 | -0.181 |
|  | *p-value* | 0.225 | 0.613 | 0.365 | 0.614 | 0.621 | 0.459 |
| **A:C ratio HID** | *Pearson’s r* | **0.579** | 0.240 | 0.344 | -0.401 | -0.140 | **0.544** |
|  | *p-value* | **0.012** | 0.322 | 0.162 | 0.088 | 0.579 | **0.016** |
